# Genetic Patterns of Common-Bean Seed Acquisition and Early-Stage Adoption Among Farmer Groups in Western Uganda

**DOI:** 10.3389/fpls.2018.00586

**Published:** 2018-05-11

**Authors:** Erin L. Wilkus, Jorge C. Berny Mier y Teran, Clare M. Mukankusi, Paul Gepts

**Affiliations:** ^1^Department of Plant Sciences, Section of Crop & Ecosystem Sciences, University of California, Davis, Davis, CA, United States; ^2^Centro Internacional de Agricultura Tropical (CIAT), Kampala, Uganda

**Keywords:** seed system, participatory variety selection, single nucleotide polymorphisms (SNP), farmer association, variety adoption, population structure, genetic diversity, *Phaseolus vulgaris*

## Abstract

Widespread adoption of new varieties can be valuable, especially where improved agricultural production technologies are hard to access. However, as farmers adopt new varieties, *in situ* population structure and genetic diversity of their seed holdings can change drastically. Consequences of adoption are still poorly understood due to a lack of crop genetic diversity assessments and detailed surveys of farmers' seed management practices. Common bean (*Phaseolus vulgaris*) is an excellent model for these types of studies, as it has a long history of cultivation among smallholder farmers, exhibits eco-geographic patterns of diversity (e.g., Andean vs. Mesoamerican gene-pools), and has been subjected to post-Columbian dispersal and recent introduction of improved cultivars. The Hoima district of western Uganda additionally provides an excellent social setting for evaluating consequences of adoption because access to improved varieties has varied across farmer groups in this production region. This study establishes a baseline understanding of the common bean diversity found among household producers in Uganda and compares the crop population structure, diversity and consequences of adoption of household producers with different adoption practices. Molecular diversity analysis, based on 4,955 single nucleotide polymorphism (SNP) markers, evaluated a total of 1,156 seed samples that included 196 household samples collected from household producers in the Hoima district, 19 breeder-selected varieties used in participatory breeding activities that had taken place prior to the study in the region, and a global bean germplasm collection. Households that had participated in regional participatory breeding efforts were more likely to adopt new varieties and, consequently, diversify their seed stocks than those that had not participated. Of the three farmer groups that participated in breeding efforts, households from the farmer group with the longest history of bean production were more likely to conserve “Seed Engufu”, a local “Calima”-type variety of the Andean bean gene pool, and, at the same time, introduce rare Mesoamerican gene pool varieties into household seed stocks.

## Introduction

Varietal adoption and ongoing selection under agronomic and social pressures represent two major processes that shape *in situ* crop genetic diversity and the generation and maintenance of distinct “landraces” (Zizumbo-Villarreal et al., 2005; Worthington et al., 2012; Pautasso et al., 2013; Soleri et al., 2013). A better understanding of these processes can support efforts to introduce new varieties without undermining the seed systems that provide for and support valuable genetic resources on-farm (*in situ*). This study investigates and compares these dynamics among household producers in Hoima, Uganda. The objective of the study is to primarily describe farmers' seed stocks in relation to breeder-selected varieties found within country and the global bean reference collection. This analysis then supports the second objective, namely to understand the effects of seed sources and early-stage adoption on seed stock population structure and genetic diversity. The analysis draws from household survey data and seed stock molecular data that span diverse groups of common bean (*Phaseolus vulgaris*)-producing households to better understand how these processes can vary across social settings. Hoima, Uganda, offered an ideal site for evaluating this topic as it represents a major bean production region for the county and was designated as a focal location for implementing participatory breeding activities in 2012, just 2 years prior to this study.

### History of common bean exchange in Uganda

Seed exchange is defined broadly here as the movement of seed from a supplier to a producer as a market transaction (formal or informal) or through the provision of freely supplied and accepted (consciously or unconsciously) seed. Common bean (*P. vulgaris*) seed exchange in Uganda, presumably began shortly after the introduction of both Andean (large seed type) and Mesoamerican (small seed type) bean domesticates in the 18th century (Gepts and Bliss, 1988). Since then, a combination of both traditional bean varieties and—since the late twentieth century—bred varieties arising from formal breeding efforts, have been cultivated in the country. Most seed exchange subsequent to this introduction occurred through informal social networks that linked households, farmer groups (Rubyogo et al., 2010), and farmers' markets (David and Sperling, 1999). The longstanding role that household farmers played in selecting beans with preferred agronomic and social traits suggests that they have had a major role in determining *in situ* bean population characteristics.

Historical production statistics indicate that the bean varieties grown in Uganda have been shaped in large part by social factors, rather than agronomic conditions alone (Johnson et al., 2003). Varietal diversity, for instance, has been associated with bean market standards; specific market preferences have contributed to the maintenance of certain seed types and varieties. For instance, production statistics from 1998 indicate that bean varietal diversity across production areas of sub-Saharan Africa was highest where common bean was marketed and consumed as a complex of varietal mixtures, namely, the Great Lakes Region (Wortmann et al., 1998). Production records suggest that household farmers in East Africa historically preferred Andean beans, and red mottled types in particular, which are similar to the Colombian “Diacol Calima” variety (Orozco et al., 1964; Voysest, 2000). Varieties with Calima-type seeds are well known for their relatively high and stable productivity under moderately good growing conditions, short cooking time, and high marketability (Wortmann et al., 1998). The most recent available estimate from 1998 found that more land in Africa was devoted to Calima bean production than any other seed type (Wortmann et al., 1998; Beebe et al., 2013). Through the ongoing processes of seed exchange and selection for these preferred large-seeded types, the Andean gene pool quickly became the predominant bean gene pool among producers while the Mesoamerican gene pool became increasingly rare (Martin and Adams, 1987a,b; Gepts and Bliss, 1988).

It is also likely that household seed production processes contributed to the generation and maintenance of novel plant material known as “landraces”. For instance, Martin and Adams (1987a,b) concluded that household seed handling practices contributed to the generation and maintenance of multiple bean landraces in Malawi. The landrace concept distinguishes the products of selection by producers under social and agronomic conditions from the products of formal breeding. Landraces have been valued for their cultural heritage and potential to serve as better parental material than cultivars (Zeven, 1998). However, very little evidence is available surrounding the contribution of landrace varieties to bean production in Uganda.

Formal breeding efforts, which include developing and disseminating new varieties, have also impacted the genetic composition of *in situ* common bean (Sperling et al., 1996; Kalyebara, 2005). In Uganda, there was a widespread adoption of K 132 (Kawomera), a CIAT-bred Calima seed type (CAL 96) with a characteristic red/white mottled pattern, after it was released in 1994 (David et al., 2000). Pedigrees of formally released varieties additionally show that a wide range of germplasm material was used by breeders in the development of novel recombinant lines. In turn, this wide range of genetic material could have had significant impacts on farmers' common bean seed stocks. However, estimates from 2005 indicate that the formal seed sector only provided 6% of the seed required to support national production levels (Almekinders and Louwaars, 2008). These assessments suggest that formally bred and released varieties have had limited impact on household seed stocks. Nevertheless, little empirical data is available to evaluate this impact as studies on varietal adoption and impacts on crop genetic diversity are lacking.

### History of participatory breeding and dissemination in Hoima, Uganda

Recent breeding and varietal dissemination efforts in Uganda are well documented and particularly relevant to the communities involved in the current study. An evaluation of Uganda production conditions from 2010 found that Hoima District in the Albertine Rift of western Uganda had highly degraded landscapes, rapidly decreasing soil fertility, and increasingly variable rainfall levels (Kristjanson et al., 2012; Mukankusi et al., 2015). Based on this characterization, Hoima was designated as a learning site for research and development of climate-change-resilient bean varieties under the Climate Change, Agricultural & Food Security (CCAFS) program of the CGIAR. In 2012, CCAFS initiated participatory varietal selection (PVS) trials that were implemented by the International Center for Tropical Agriculture (CIAT, Cali, Colombia) through the Pan-Africa Bean Research Alliance (PABRA), in collaboration with the National Agricultural Research Organization (NARO) and household producers from Kyabigambire sub-county of Hoima District. The purpose of the trials was to identify preferred varieties from CIAT plant breeding programs based on household preferences and performance under household growing conditions. CIAT provided household producers with a set of breeder-selected varieties for PVS trials that households then grew and evaluated (Table 1).

**Table 1:** Morphological characteristics and breeding and release histories of CIAT-selected varieties.

	**Name**	**Gene pool**	**Race**	**Varietal release history**	**Genealogy**	**Seed area (mm^2^)**	**Seed weight (g\100)**	**Seed length (mm)**	**Circularity**
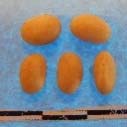	NABE 6 (UBR92)	Mesoamerican	Mesoamerica	1999, not included in PVS	Selection on local varieties or landraces	39.33 (8.02)	52.63	8.89 (1.64)	0.67 (0.04)
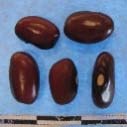	NABE 3 (MCM2001)	Mesoamerican	Mesoamerica	1995, not included in PVS	IVT831607 × RAB 71	55.67 (4.76)	25.5	10.56 (0.55)	0.76 (0.02)
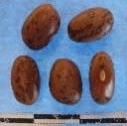	K 131 Kabalira\ (MCM5001)	Mesoamerican	Mesoamerica	1994 CIAT-release not included in PVS	IVT831629 × BAT 1554	55.36 (4.13)	29.36	10.27 (0.43)	0.79 (0.02)
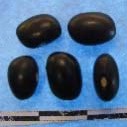	NABE2 (MCM1015)	Mesoamerican	Mesoamerica	1995 CIAT-release, PVS phase 2	IVT831629 × BAT 1554	67.29 (7.80)	34.14	11.84 (0.80)	0.78 (0.02)
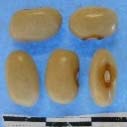	Roba 1	Mesoamerican	Durango-Jalisco	PVS Phase 1 and 2	G 7951 × A 30	71.57 (6.69)	34.0	11.65 (0.62)	0.80 (0.02)
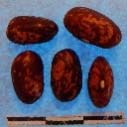	NABE 17	Andean	Nueva-Granada	PVS Phase 2	Kanyebwa × AB136	77.36 (11.82)	33.5	12.75 (1.21)	0.75 (0.03)
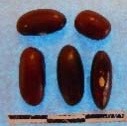	KATX 56	Andean	Nueva-Granada	1995 KARI-release, PVS phase 1 and 2	Selection on local varieties or landraces	70.61 (9.22)	36.85	13.57 (1.23)	0.68 (0.03)
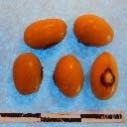	KATB 1 (Kat-Bean 1/ katheka)	Andean	Nueva-Granada	1987 KARI-release, PVS phase 1 and 2	Selection on local varieties or landraces	58.55 (7.06)	20.31	10.54 (0.71)	0.77 (0.06)
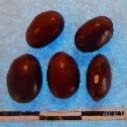	KATB 9 (Kat-Bean 9)	Andean	Nueva-Granada	1998 KARI-release, PVS phase 1 and 2	Selection on local varieties or landraces	57.99 (8.05)	38.89	10.66 (0.88)	0.77 (0.03)
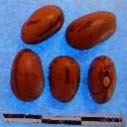	NABE 15	Andean	Nueva-Granada	PVS Phase 1 and 2	Kanyebwa/ P1 207262	71.87 (8.78)	35.71	12.06 (0.93)	0.77 (0.02)
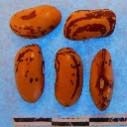	NABE 11 (AFR 721)	Andean	Nueva-Granada	Not included in PVS		95.92 (9.80)	44.13	15.31 (1.04)	0.71 (0.03)
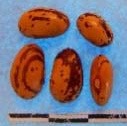	NABE 21	Andean	Nueva-Granada	PVS Phase 2	Kanyebwa × PI 207262	69.53 (8.11)	38.06	11.98 (0.80)	0.77 (0.02)
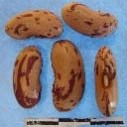	NABE 5 (SUG 73)	Andean	Nueva-Granada	1999, c	AFR 88 × AFR 199	130.88 (37.52)	85	17.66 (4.74)	0.67 (0.04)
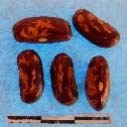	KI32 (Kipopo 1;kawonera;CAL96)	Andean		1994 CIAT-release, PVS phase 2	Calima-2 × Argentino1	132.25 (14.80)	77.77	18.66 (1.06)	0.65 (0.02)
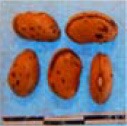	NABE 12C	Andean	Nueva-Granada	Not included in PVS trials		75.57 (8.26)	43.26	12.28 (0.81)	0.77 (0.03)
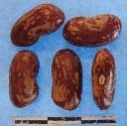	NABE 4 (POA2)	Andean	Nueva-Granada	1999, not included in PVS	SUG 47 × CAL 103	124.36 (6.40)	67.69	17.18 (0.55)	0.68 (0.04)
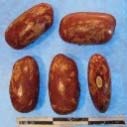	NABE 1	Andean		1995 CIAT-release, PVS phase 1 and 2	G 21724 × G 7385	107.67 (5.54)	60.48	15.30 (0.54)	0.72 (0.03)
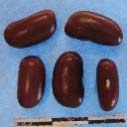	Drk 70	Andean	Nueva-Granada	1999, not included in PVS	PVA 7 × AFR 169	101.49 (6.96)	68.05	15.02 (0.70)	0.73 (0.02)
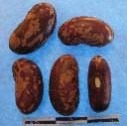	K20 (Nambale)	Andean		1970, PVS Phase 1 and 2		128.98 (8.30)	79.23	17.46 (0.66)	0.68 (0.02)

Participatory Variety Selection (PVS) activities were implemented over the course of two phases. Phase 1 took place during the first crop season of 2012 (March–July) and Phase 2 took place during the first and second seasons of 2013 (March–July and August–December). Households were trained in recommended planting techniques and given small quantities (150 g) of the breeder-selected varieties at the end of each season following evaluations. Households could multiply these small quantities and maintain them within household seed stocks for future household bean production.

The study presented here complements the PVS activity by assessing the contents of household seed stocks subsequent to household participation in PVS trials. Molecular methods were used to compare the population structure and genetic diversity of organization-sourced, market-purchased, and household-saved common bean seed among household producers. In order to evaluate the role of seed exchange, the analysis was conducted across households belonging to different seed exchange networks based on breeding program-affiliation and farmer group membership. Breeding-program affiliation and farmer-group membership reflected different levels of access to seed varieties from formal-sector breeding efforts and variation in community-level preferences, respectively.

## Materials and methods

The overall research strategy was as follows. A baseline understanding of household seed stock composition and structure was established through population STRUCTURE analysis and phylogenetic reconstruction of a bean sample comprising the entire household seed collection, a global common bean germplasm collection, and specific breeder-selected varieties. In a second stage, the same analytical methods were applied to compare household seed stock from different sources and determine the prevalence of adopted materials and the impact of early-stage adoption on seed stock population structure and diversity. We define early-stage adoption as the process of acquiring new varieties and incorporating them into household seed stock. Adopted material was considered any farmer-identified variety, that was purchased or received freely from outside of the household and was present in household seed stock during the survey period. Adoption patterns are described on the basis of the addition of outside seed materials to the household seed stock, their genetic identity and seed source, and the consequences on the overall genetic characteristics of that seed stock.

To evaluate the role of seed exchange networks on adoption patterns, the results from households that participated in PVS trials (breeding program-affiliated) were compared to those from the group of households that had not participated in PVS trials (breeding-program-unaffiliated). Thirdly, this comparison was performed across three breeding program-affiliated farmer groups to determine if differences existed at the community level.

### Household selection

The study began with purposive and stratified selection of households with historically distinct forms of participation in regional- and community-level seed exchange networks. A total of 82 households were surveyed where 41 of the households had participated in the PVS trials (breeding-program-affiliated) and 41 households had not participated in the PVS trials (breeding-program-unaffiliated). The 41 breeding-program-affiliated households were composed of three farmer groups, namely, Akumulikire Women Cooperative with 11 households, Kakindo Sustainable Cooperative with 13 households, and Kyamaleera Handcraft Cooperative with 17 households.

Households from the Kyamaleera Handcraft Group had the largest average household (7 ± 2) of the three farmer groups. Most households from this group indicated that they formed the farmer group to increase seed production, market information, and seed exchange. Households from Kakindo Sustainable Cooperative expressed a passion for maintaining diversity and traditional varieties in their seed stocks. These households had the smallest average household size (5 ± 2) of the three farmer groups. The heads of households from the Kakindo Sustainable Cooperative also tended to be older (31 ± 10) and have more bean production experience than the other farmer groups. Households from the Akumulikire Women Group reported that they were primarily motivated by commercial production goals. Household heads from Akumulikire Women Group tended to be younger (21 ± 5) and had less bean production experience than the other farmer groups.

None of the breeding program-affiliated households were members of more than one farmer group. The households spanned all parishes within Kyabigambire sub-county of Hoima District except for one parish that was not included in the PVS trials (Kisabagwa parish). Breeding program-unaffiliated households were randomly selected across the same Kyabigambire parishes and neighborhoods as breeding program-affiliated households.

### Plant material

The collection included a set of 196 household samples collected from producers in Hoima district, 19 breeder-selected varieties (Table 1) originating from either the CIAT-Kawanda, Uganda, or CIAT-Cali, Colombia, germplasm collections. For comparison, this study included a database of a world-wide reference germplasm collection that consisted of 502 accessions of the Andean Diversity Panel (Cichy et al., 2015), 363 accessions of the USDA core collection (McClean et al., 2012; S. Kuzay, P. Hamilton-Conaty and P. Gepts, unpubl. results), and 57 reference and commercial cultivars. A subset of the breeder-selected samples included in the analysis had been evaluated and made available to breeding program-affiliated households through the CIAT-managed PVS trials from 2012 to 2013. Within the breeder-selected varieties, NABE 11, 15, 17, and 21 were bred locally by the national Ugandan bean program using CIAT-bred lines while the remaining NABE lines were bred under CIAT-led programs. The KAT lines had been bred locally by a Kenyan breeder at the Katumani Research Station in the 1990's.

The 196 household seed stock samples were collected from eight-two households between May and June of 2014, within 2–3 weeks of the first harvest since PVS trials were completed. For an exhaustive survey of household seed stock, households were asked to provide all samples from household storage and any samples that were still being processed from the most recent harvest. Households were then asked to divide seed stock into samples that they considered distinct and provide 20 seeds of each sample. Households then completed a seed survey that indicated the seed source type (organization-sourced, household-saved, or market-purchased), source distance, and year of acquisition for each sample. Most of the household seed stocks were a combination of household-saved seed, seed purchased from local markets, and newly adopted materials that were introduced through the CIAT-managed participatory breeding activities.

The multiple forms of seed exchange networks in which these households participated and the mixed sources of common bean found in household seed stocks provided an experimental system to reveal the conditions that impacted adoption patterns and consequences for *in situ* population structure and diversity. The length of time that adopted material had been present within the household seed stock varied from less than a month to multiple decades, with market-purchased varieties dating back to the 1970s. Most adopted material—under our definition—had been included in at least one season of production. Given the short time span between PVS participation and the seed surveys, the majority of adopted material was acquired and incorporated into household seed stock within the last 2 years. Therefore, these findings largely reflect outcomes of early-stage adoption, rather than long-term consequences. All the organization-sourced seed samples among affiliated households were sourced from CIAT. In contrast, the two organization-sourced samples found among -unaffiliated household seed stock were received from the National Agricultural Advisory Services (NAADS) and a “group of women friends.”

### Seed morphology

Seed morphology was evaluated with SmartGrain, a high-throughput phenotyping software (Tanabata et al., 2012). Twenty seeds of each sample were scanned and analyzed to obtain average seed length, width and circularity measurements. Seed length (L) is defined as the maximum distance between points on the seed perimeter (P) and was determined by calculating all segment distances between all pairs of points on the perimeter (Suzuki and Abe, 1985). Seed width is defined as the longest segment that is perpendicular to the length segment. Seed circularity is calculated as: $CS=\;\frac{4\pi \left(AS\right)}{PL^{2}}$, where AS is the seed area computed as the area within the set of seed perimeter coordinates that the SmartGrain image analysis software generates based on the border algorithm developed by Suzuki and Abe (1985). PL is the perimeter length, which is similarly calculated by the SmartGrain image analysis software. Seed weight was taken as the average weight of all the seeds from each sample. A 100-count seed weight estimate was derived to standardize the seed weight measurement.

### Greenhouse protocol and DNA extraction

The entire collection of household seed samples was planted in planting tubes three times in triplicate in the UC Davis Greenhouse #75 on August 14, 2014, and grown under standard greenhouse management conditions. Leaf samples were collected at the first trifoliolate stage at 2–3 weeks from planting, when cotyledon leaf and the first true leaf were fully developed. Harvested leaf tissue was immediately placed in microcentrifuge tubes and stored on dry ice in a portable cooler. Nine seeds of each sample were randomly selected and bulked for SNP-based genotyping, population structure analysis and phylogenetic reconstructions. DNA was extracted from lyophilized leaf tissue using Qiagen DNeasy Plant Kit. DNA was extracted directly from seed using an adjusted Cetyl Trimethyl Ammonium Bromide (CTAB) protocol when samples did not germinate in the greenhouse. The DNA quality was evaluated on 1% agarose gels and quantified with QUANTITY ONE v. 4.0.3 software (Bio-Rad Lab., Hercules, CA). DNA samples were then diluted to a standard concentration of 5 ng/μl for sequencing.

### SNP-based genotyping

The Illumina Infinium “BeadChip BARCBean6K-3” (Song et al., 2015) from the USDA National Institute of Food and Agriculture BeanCAP Project (Grant number 2009-01929) was used to genotype the entire seed collection. Single nucleotide polymorphism (SNP) genotyping was conducted courtesy of Dr. Perry Cregan, USDA-ARS, Soybean Genomics Improvement Laboratory, BARC-West, Beltsville, MD, on the Illumina platform following the Infinium HD Assay Ultra Protocol (Illumina, San Diego, CA). Sequencing output of the BARCBean6K-3 BeanChip was evaluated using GenomeStudio software. Clusterw2 software was used to align sequences and generate SNP calls. In order to generate reliable SNP calls for household seed samples and breeder-selected samples, cluster files were calibrated from the default set of cluster files. 4,955 of the 5,398 single-nucleotide polymorphism (SNP) markers were then called using the new cluster files with a Gencall score cutoff of 0.15, according to the GenomeStudio Genotyping Module v1.8.4 (Illumina, San Diego, CA). SNP data of samples from the global germplasm collection BARCBean6K-3 assay were filtered to include only those having less than 5% missing data and 5% heterozygosity. SNP data of the remaining samples were pruned to 1,870 markers to reduce linkage disequilibrium. Pruning was performed in a moving window of 50 SNPs removing one of a pair of SNPs if the linkage disequilibrium was higher than 0.6. The steps of filtering and pruning were performed in PLINK (Purcell et al., 2007). The SNPs identified as part of this research in the African beans and the USDA common-bean core collection were deposited in the University of California Dash Repository: https://doi.org/10.25338/B8CS3Z. The SNPs identified in the Andean Diversity Panel (Cichy et al., 2015) are available from http://arsftfbean.uprm.edu/bean/?p=472.

### Population structure

A principal component analysis was performed with adegenet (Jombart and Ahmed, 2011). Population structure was evaluated based on SNP data using a model-based approach implemented in the STRUCTURE software (Pritchard et al., 2000). The estimated proportion of each cluster within an individual sample (q) was calculated for K ranging from 2 to 12 populations with 10 runs for each K-value. The analysis was conducted with a burn-in period of 100,000 and 100,000 iterations for estimating the parameters. The criterion suggested by Evanno et al. (2005) based on second-order rate of change in the log probability of data between successive K-values, was used to determine the most likely number of clusters (K). Seed samples were assigned to populations based on a membership coefficient cutoff of 0.75.

### Phylogenetic reconstruction

The Ade4 and adegenet R software packages were used for generation of genetic distance-based neighbor-joining trees. Two input files were used to construct neighbor-joining trees: a FASTA file containing SNP data and an annotation file containing seed source, household breeding program-affiliation and farmer group-membership. SNP data was converted into a FASTA file format manually using FASTA code conventions. The annotation file was a comma-separated file. Genealogical reconstruction began with computing genetic distances (Supplementary Table 1) using the Tamura and Nei (1993) model. In order to increase the robustness of the phylogenetic reconstruction, bootstrap percentages (BP) were calculated for each node after 1000 re-samplings (Felsenstein, 1985) and all nodes with bootstrap values <70%, were collapsed. The estimated genetic distances between CIAT-sourced household varieties and breeder-selected material were used to infer the breeder-selected variety that the adopted material represented, i.e., the potential source variety. A genetic distance of less than 0.1 was established as the confidence threshold based on the observed range of genetic distance values.

### Relative abundance of adopted seed and contribution to crop diversity

*T*-tests and analysis of variance (ANOVA) compared the relative abundance of organization-sourced, market-purchased, and household-saved Andean and Mesoamerican seed samples between program-affiliated and -unaffiliated households and across farmer groups. The genetic diversity and analysis of molecular variance (AMOVA) were performed in GenAlEx version 6.5 (Peakall and Smouse, 2012). Genetic diversity was calculated as: *SSWP x* (*n*−1)−1, where SSWP = Within-population sum of squares and n = number of samples. Analysis of molecular variance (AMOVA) was conducted to evaluate subpopulation structure based on genetic distances between samples. AMOVA analyses partitioned total molecular variance within and across seed sources, within and between breeding program-affiliated and -unaffiliated households, and within and across farmer groups to evaluate the percent of seed stock variability explained by seed source, breeding program-affiliation and farmer group-membership. Significance was calculated using 16,000 permutations.

## Results

Population structure analysis, phylogenetic reconstruction, and diversity assessments, based on SNP data of the household collection and a broad set of global and specific breeder-selected samples are presented to demonstrate how representative household seed stocks were of the known worldwide germplasm diversity of domesticated common bean. *T*-tests and analysis of variance (ANOVA), based on the prevalence of organization-sourced material within household seed stock, are then reviewed to compare adoption levels between breeding program-affiliated and -unaffiliated households and across farmer groups. Results from population STRUCTURE and phylogenetic reconstruction methods demonstrate consequences of adoption for seed stock composition and diversity.

### Farmer household seed stock representativeness: a comparison of household samples to a global germplasm collection

**Figure 1:**
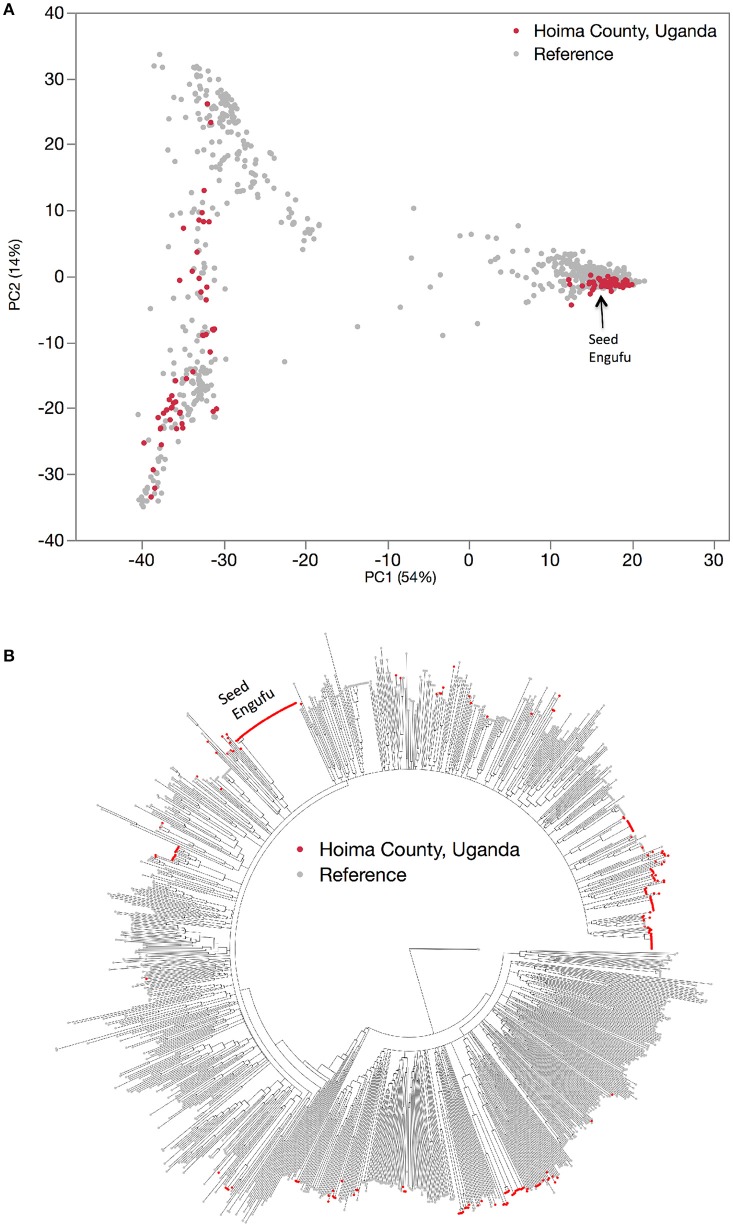
Hierarchical organization of genetic relatedness of the entire collection of reference varieties (gray) and household seed samples (red). Principal component analysis **(A)** and Neighbor joining tree **(B)** based on 4,955 Single Nucleotide Polymorphism (SNP) sites.

**Figure 2:**
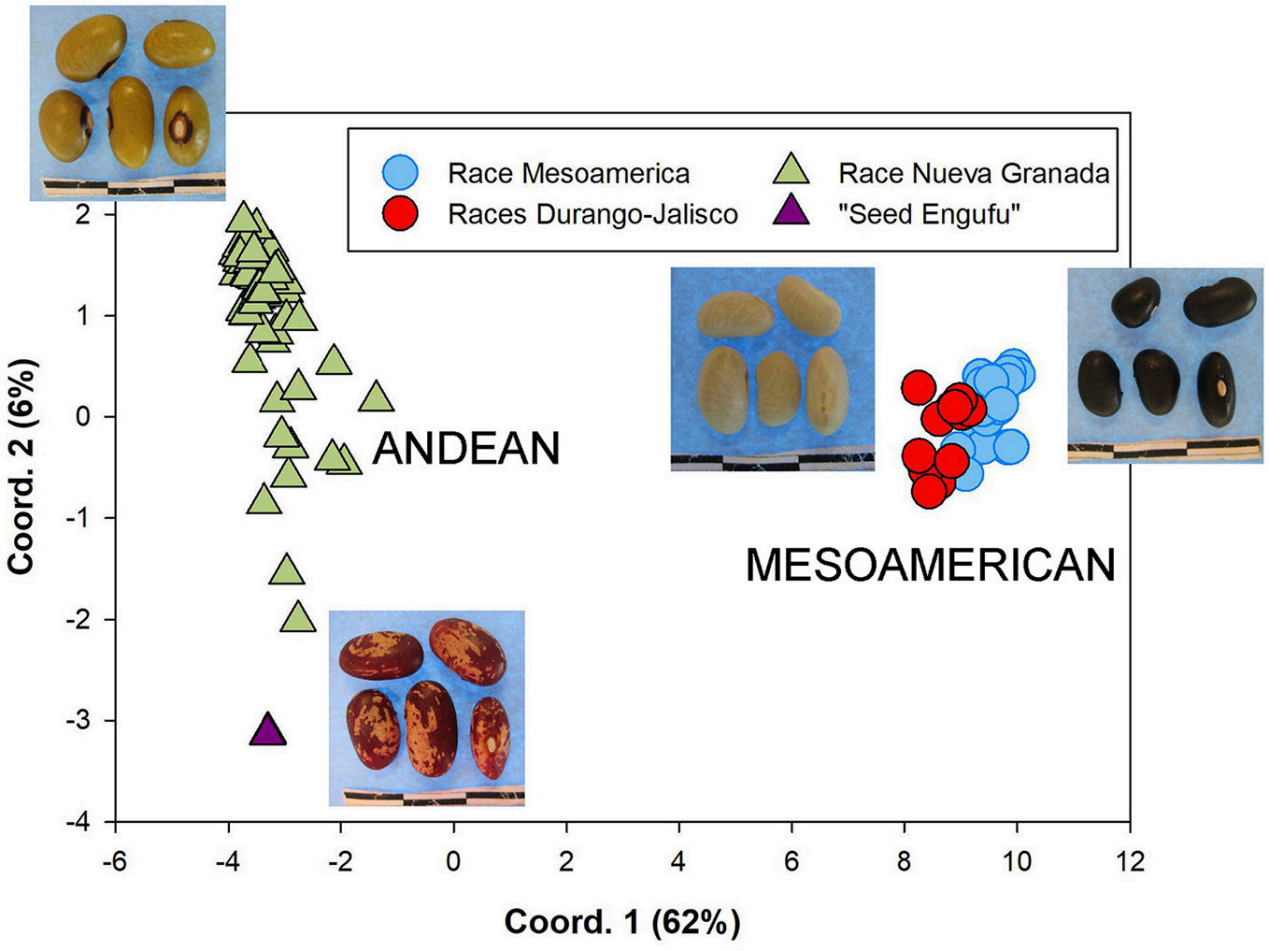
Principal coordinate analysis based on Nei's genetic distances computed among the complete collection of breeder-selected varieties and household seed samples. Each sample is represented according to membership into the *K* = 4 groups identified by STRUCTURE namely, the Andean genepool (triangle), which includes races Nueva Granada (green) and “Seed Engufu” (purple), and the Mesoamerican (circle symbols) genepool, which includes the Durango-Jalisco complex (red) and race Mesoamerica (blue).

The genetic diversity of the household seed stock was compared to that of both a global germplasm collection (Figure 1), as well as that of the collection of breeder-selected varieties (Figure 2). All three samples included materials representing the Andean and Mesoamerican genepools. The Principal Component Analysis (PCA) of the global germplasm collection generated two main clusters along the first Principal Component (PC1, 54%), one composed of Andean gene pool samples (higher PC1 coordinate values) and the other representing Mesoamerican gene pool samples (lower PC1 coordinate values, Figure 1A). The Mesoamerican gene pool was further subdivided in two clusters along PC2 (14%) representing two distinct races: Durango-Jalisco (positive values along PC2) and Mesoamerican (negative values along PC2). Similarly, the PCA of the household samples (Hoima district) and the collection of breeder-selected varieties identified two main clusters representing the Andean (lower PC1 value coordinates, 62%) and Mesoamerican (higher PC1 value coordinates) genepools (Figure 2). Control samples and visual inspection of samples further confirmed that the two populations within the Mesoamerican gene pool represented the Durango-Jalisco complex (G2400) and race Mesoamerica (G1975), and one of the populations within the Andean gene pool represented race Nueva Granada (G5708).

**Figure 3:**
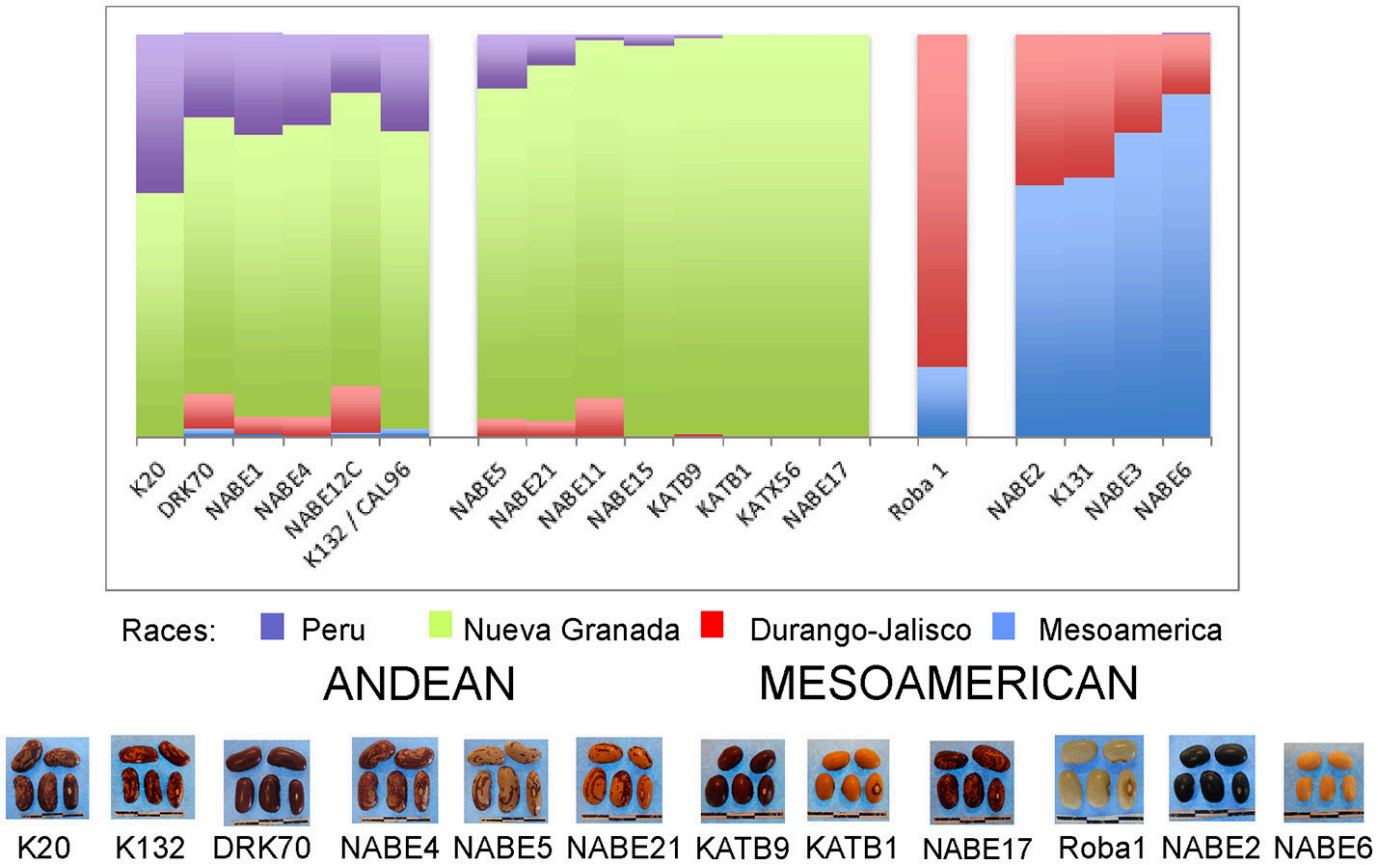
Hierarchical organization of genetic relatedness of the CIAT-selected varieties based on 4955 Single Nucleotide Polymorphism (SNP) sites and analyzed using STRUCTURE software as described in section Materials and Methods for *K* = 4. Each color represents a distinct common bean cluster within either the Andean (race Nueva Granada: green and “Seed Engufu”: purple) or Mesoamerican (Durango-Jalisco complex: red; race Mesoamerica: blue) genepools. Each bar represents a seed sample and the lengths of each colored segment represents the proportions of each sample assigned to the four clusters. Photographs of a representative subset of reference varieties reveal morphological similarities and distinctions among reference samples.

**Figure 4:**
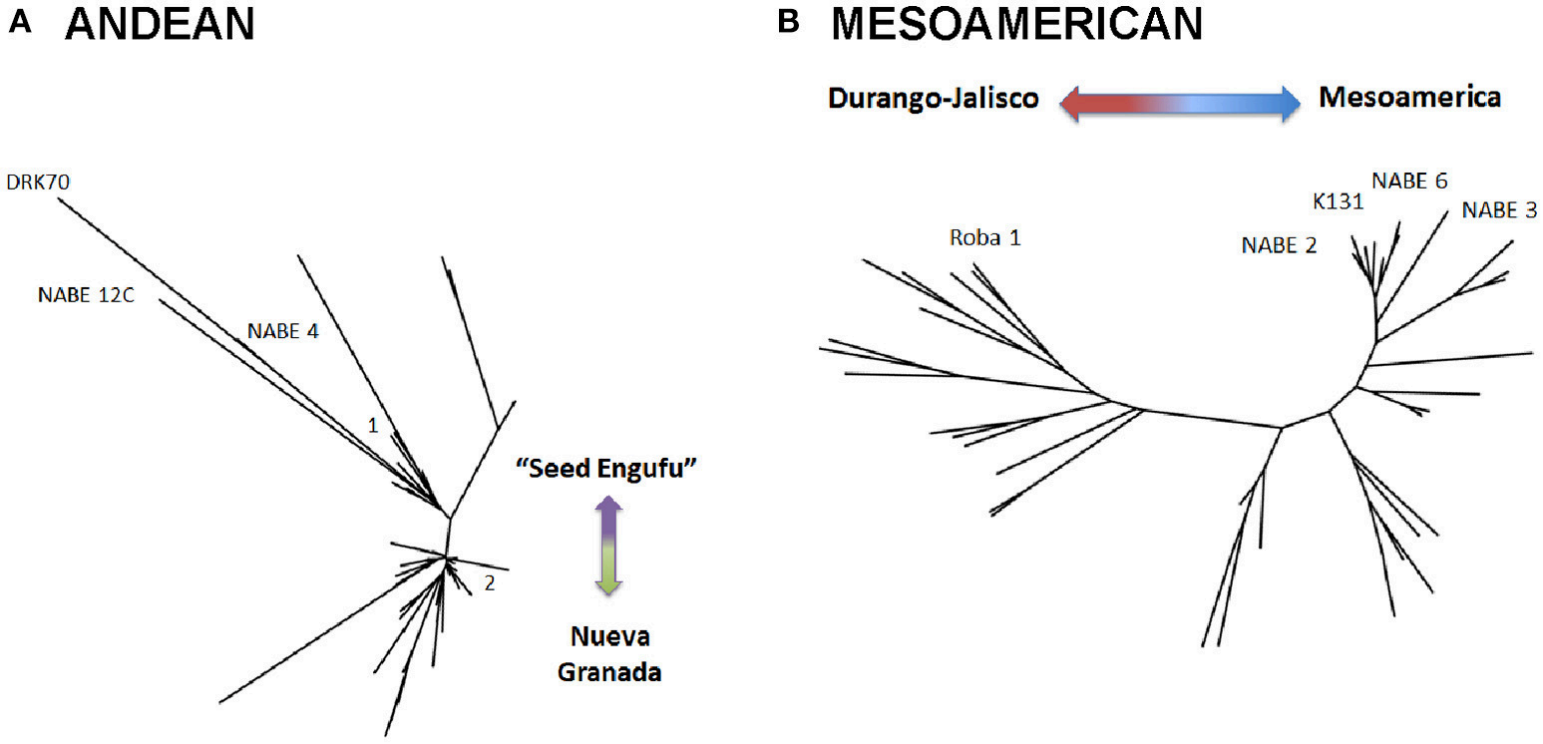
Neighbor-joining trees of single nucleotide polymorphism (SNP) diversity of Andean **(A)** and Mesoamerican **(B)** samples based on Nei's genetic distance. Bootstrap percentages (BP) were calculated for each node after 1,000 resamplings and all nodes with bootstrap values 70%, were collapsed. The trees depict the relatedness of reference varieties within the entire collection of reference and household seed samples. The Andean tree **(A)** depicts reference varieties on two main clades, 1 and 2. Clade 1 did not contain a control genotype that indicates the race associated with samples on this Clade. Clade 2 represents race Nueva Granada. The Mesoamerican tree **(B)** depicts three distinct clades. The leftmost clade represents the Durango-Jalisco race, the right of center clade represents race Mesoamerica and the upper right clade include additional Mesoamerican race varieties.

A STRUCTURE analysis (Pritchard et al., 2000) classified 14 out of the 19 breeder-selected varieties as Andean (Figure 3). The Andean breeder-selected varieties occupied two closely related clades in the Andean neighbor-joining tree (Figure 4A). K 20, K 132 and NABE 1 were located on Clade 1 of the Andean neighbor-joining tree. The remaining majority of breeder-selected varieties (NABE 11, NABE 21, KATB 9, NABE 15, KATB 1, NABE 5, NABE 17, KATX 56) were located with the race Nueva Granada control genotype (G5708) on Clade 2. NABE 12C, DRK 70 and NABE 4 were most dissimilar from the other Andean breeder-selected varieties as indicated by their longer branch lengths. Breeder-selected Mesoamerican varieties were located on two of the three distinct clades on the Mesoamerican NJ tree containing breeder-selected accessions with samples from the household collection (Figure 4B). The leftmost clade contained the Durango-Jalisco control genotype (G2400), and the reference variety, Roba 1. Of the two more closely related clades, one contained the control genotype of race Mesoamerica (G1975) and the other, rightmost clade exclusively contained the remaining Mesoamerican breeder-selected varieties (NABE 2, NABE 3, NABE 6 and K 131).

**Figure 5:**
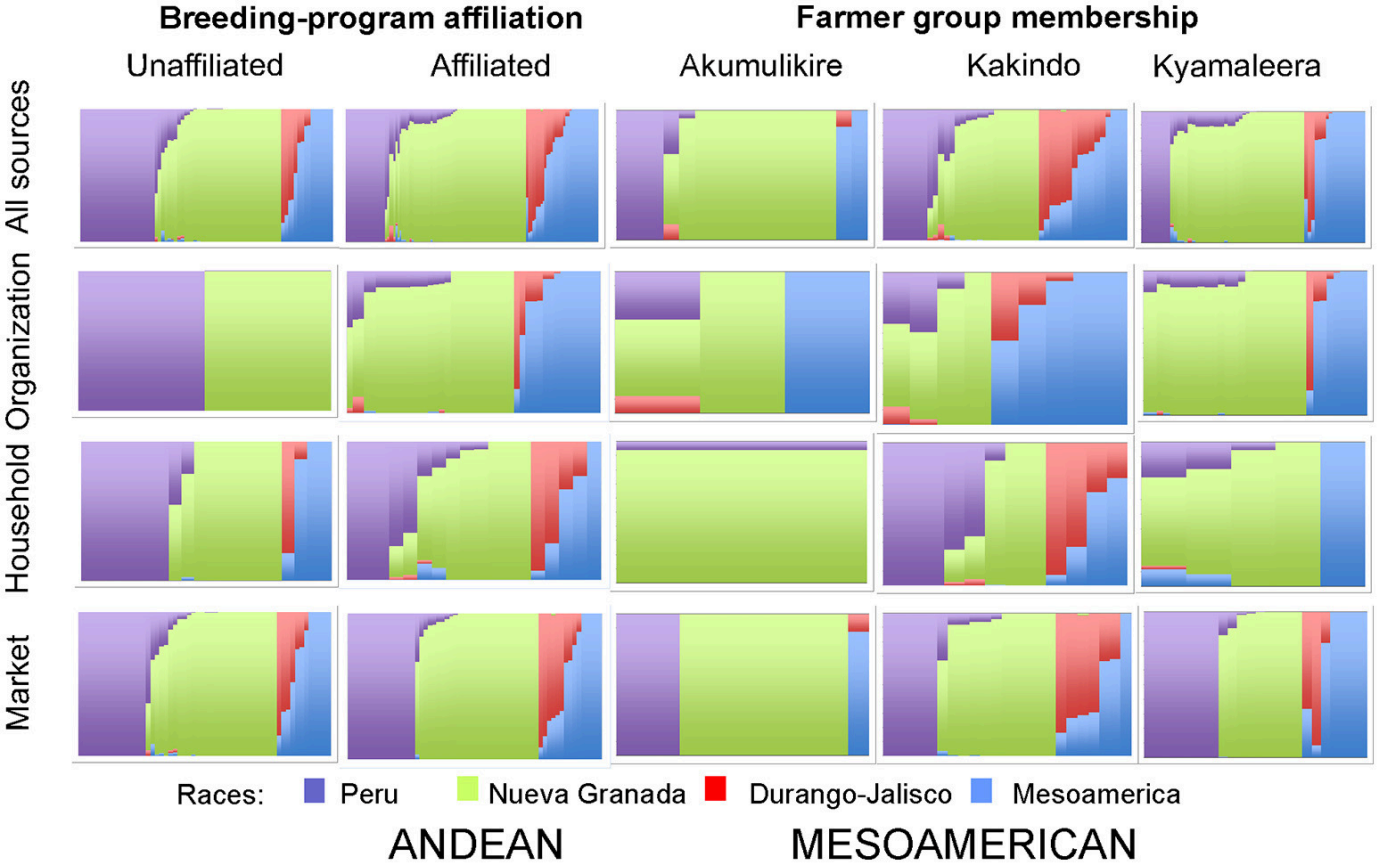
Hierarchical organization of genetic relatedness of household seed samples across seed sources, between breeding-program affiliated and unaffiliated households and across farmer groups based on 4,955 Single Nucleotide Polymorphism (SNP) sites and analyzed using STRUCTURE software as described in section Materials and Methods for *K* = 4. Each color represents a distinct cluster within either the Andean and Mesoamerican genepools. The clusters represent Andean race Nueva Granada (NG: green) and “Seed Engufu” (SE: purple) or the Mesoamerican complex Durango-Jalisco (DJ: red) and race Mesoamerica (M: blue). Each bar represents a seed sample and the length of the colored segment represents the proportion of each race to the genetic composition of the sample.

With one exception, the PCA analyses (Figures 1A, 2) and neighbor joining trees (Figures 1B, 4A) did not detect any Andean household samples that were distinct from germplasm found in the global collection. The exception was revealed by the STRUCTURE analysis of the breeder-selected and household collection samples, which identified a fourth distinct population, based on the Evanno et al. (2005) test, indicating more complex population structure in household seed stocks relative to the global germplasm collection (Figure 5). Control genotypes, visual inspection of seed morphological traits, and prior information obtained from previous molecular analyses (Gepts, 1988; Ddamulira et al., 2014; Okii et al., 2014), provided additional empirical support for characterization of the *K* = 4 populations. The fourth population was primarily composed of Andean seed samples that household members consistently referred to as “Seed Engufu” (translated from the local Bantu language, Runyoro or Nyoro, as “short seed”). Households provided variants of the Seed Engufu identifier for a small fraction of the distinct samples. The neighbor-joining tree representation of household samples and the global germplasm collection characterized Seed Engufu as a distinct and mostly monomorphic clade (Figure 1B). Four samples from the Andean Diversity Panel, all of which originated from eastern Africa, were represented in the same clade as Seed Engufu (Rozi Koko, PI449428, ADP751, and ADP758, Figure 1B).

The Seed Engufu samples formed a monomorphic group based on morphological characteristics and SNP data. Morphological evaluations confirmed that the seed samples that were assigned to STRUCTURE population *K* = 4 represented a morphologically distinct seed type. The seeds were characteristically a short, red mottled Calima-type seed. The majority of breeder-selected samples showed evidence of low levels of introgression that reflect hybridization events between the Seed Engufu cluster, race Nueva Granada and the Durango-Jalisco race complex of the Mesoamerican gene pool (Figure 3).

Household samples of Mesoamerican seed represented a relatively broad range of the race Mesoamerica germplasm found in the global collection (Figure 1A) as well as accessions that were intermediate between race Mesoamerica and race Durango-Jalisco. However, Durango-Jalisco was underrepresented among household producers in Hoima. Only two household samples were clustered with the global collection of Durango-Jalisco samples.

### Adoption patterns among farmer groups

Breeding program-affiliated households tended to show higher levels of adoption relative to unaffiliated households [Table 2: 27 vs. 3%, *t*_(81)_ = 4.1264, *p* < 0.0001]. Adoption patterns of households from one of the breeding program-affiliated groups (Kyamaleera Handcraft Group) were most distinct from-unaffiliated households and the remaining breeding program-affiliated farmer groups, based on the uniquely high adoption levels of Andean material among those households: 35 vs. 9% (Akumulikire), 6% (Kakindo), and 3% (unaffiliated, Table 2).

**Table 2:** Composition of household seed stock expressed as the proportion of total household seed stock.

	**Between affiliated and unaffiliated households**			**Across affiliated farmer groups**			
	**Unaffiliated mean (SE)^a^**	**Affiliated mean (SE)**	**T-stat**	**Akumulikire mean (SE)**	**Kakindo mean (SE)**	**Kyamaleera mean (SE)**	**F-ratio**
*N_*t*_*^b^	78	118		16	42	59	
A	0.83 (0.27)	0.78 (0.28)	−0.7954	0.95 (0.15)	0.67 (0.29)	0.76 (0.28)	3.78^*^
M	0.16 (0.26)	0.22 (0.28)	0.8680	0.05 (0.15)	0.33 (0.29)	0.24 (0.28)	3.78^*^
**ORGANIZATION**							
All	0.03 (0.16)	0.27 (0.34)	4.1264^*^	0.14 (0.32)	0.12 (0.17)	0.48 (0.36)	6.49^*^
A	0.03 (0.16)	0.19 (0.28)	3.1822^*^	0.09 (0.20)	0.06 (0.13)	0.35 (0.33)	6.11^*^
M	0.00 (0.00)	0.04 (0.19)	2.8352^*^	0.05 (0.15)	0.06 (0.12)	0.13 (0.25)	0.72
**HOUSEHOLD**							
All	0.24 (0.40)	0.09 (0.18)	2.1854^*^	0.05 (0.15)	0.15 (0.24)	0.06 (0.14)	1.22
A	0.20 (0.37)	0.07 (0.16)	2.1488^*^	0.05 (0.15)	0.11 (0.19)	0.05 (0.13)	0.74
M	0.03 (0.10)	0.02 (0.06)	0.6972	0.00 (0.00)	0.03 (0.09)	0.01 (0.04)	1.33
**MARKET**							
All	0.73 (0.42)	0.64 (0.37)	1.0649	0.82 (0.34)	0.73 (0.32)	0.46 (0.36)	4.36^*^
A	0.60 (0.42)	0.52 (0.40)	0.8035	0.82 (0.34)	0.49 (0.31)	0.36 (0.40)	5.65^*^
M	0.14 (0.26)	0.12 (0.20)	0.3846	0.00 (0.00)	0.24 (0.25)	0.10 (0.16)	5.50^*^
**ACROSS SEED SOURCES, F-STATISTIC**							
All	45.16^*^	34.40^*^		24.43^*^	24.36^*^	10.01^*^	
A	53.97^*^	51.34^*^		49.85^*^	19.22^*^	15.65^*^	
M	7.43^*^	7.49^*^		0.67	6.31^*^	3.46^*^	

*Andean (A) and Mesoamerican (M) seed were aggregated and evaluated as distinct sub-populations for seed from all sources combined and each source separately. T-tests and analysis of variance (ANOVA) compared the relative abundance of organization-sourced, market-purchased, and household-saved Andean and Mesoamerican seed samples between program-affiliated and -unaffiliated households and across farmer groups*.

a SE, Standard Error;

b *N_t_, Total number of seed samples*.

“*” *means significance level of p < 0.05*.

*Numbers in bold: values that are significantly different between affiliated and unaffiliated households or across affiliated farmer groups*.

All of the CIAT-sourced Andean samples were assigned a potential breeder-selected source variety with a reasonable degree of confidence (genetic distance of less than 0.1). The most common potential breeder-selected source Andean varieties among the affiliated-households where NABE15 and NABE17 (Table 3). The Kyamaleera Handcraft Group housed the broadest array of potential breeder-selected sourced Andean varieties of all three-affiliated farmer groups. NABE15 and NABE17 represented 35 and 30% of the 23 CIAT-sourced samples from the Kyamaleera households. NABE 21, KATB1, KATB9, KATX56, and CAL96 were also potential breeder-selected source Andean varieties among the CIAT-sourced samples in the Kyamaleera group (Table 3). NABE 15 and NABE 17 and KATB1 were similarly represented among the CIAT-sourced samples in the Kakindo households. In addition to those varieties that were also identified in the Kyamaleera, NABE1 was present in the Kakindo CIAT-sourced samples. NABE 17 and NABE 1 were present in the Akumulikire CIAT-sourced samples.

**Table 3:** Assignment of potential breeder-selected seed source variety to household CIAT-sourced variety.

**Genepool**	**Farmer group**	**CIAT-sourced household seed sample ID**	**Adoption year**	**Potential source variety**	**Genetic distance^a^**
Andean	Kyamaleera	1	2013	NABE15	0.00
		2	2014	NABE15	0.00
		3	2013	NABE15	0.00
		4	2012	NABE15	0.00
		5	2014	NABE15	0.00
		6	2014	NABE15	0.00
		7	2014	NABE15	0.00
		8	Unknown	NABE15	0.00
		9	2012	NABE17	0.00
		10	2014	NABE17	0.00
		11	2013	NABE17	0.00
		12	2013	NABE17	0.00
		13	2012	NABE17	0.00
		14	2014	NABE17	0.00
		15	2013	NABE17	0.00
		16	2014	NABE21	0.00
		17	2010	KATB1	0.02
		18	2012	KATB1	0.00
		19	2010	KATB9	0.00
		20	2014	KATB9	0.00
		21	2014	KATX56	0.00
		22	2014	CAL96	0.01
		23	2014	CAL96	0.01
	Kakindo	24	2011	NABE15	0.00
		25	2013	NABE17	0.00
		26	2013	KATB1	0.01
		27	2011	NABE1	0.05
	Akumulikire	28	2013	NABE17	0.00
		29	2013	NABE1	0.05
Mesoamerican	Kyamaleera	30	2012	NABE2	0.05
		31	2014	NABE2	0.06
		32	2014	Roba1	0.00
		33	2014	K131	0.00
	Kakindo	34	2011	NABE2	0.04

a *Genetic distance estimates were extracted from the genetic distance matrix that was generated with the Ade4 and adegenet R software packages based on SNP data. A genetic distance of less than 0.1 was established as the confidence threshold based on the observed range of genetic distance values*.

**Figure 6:**
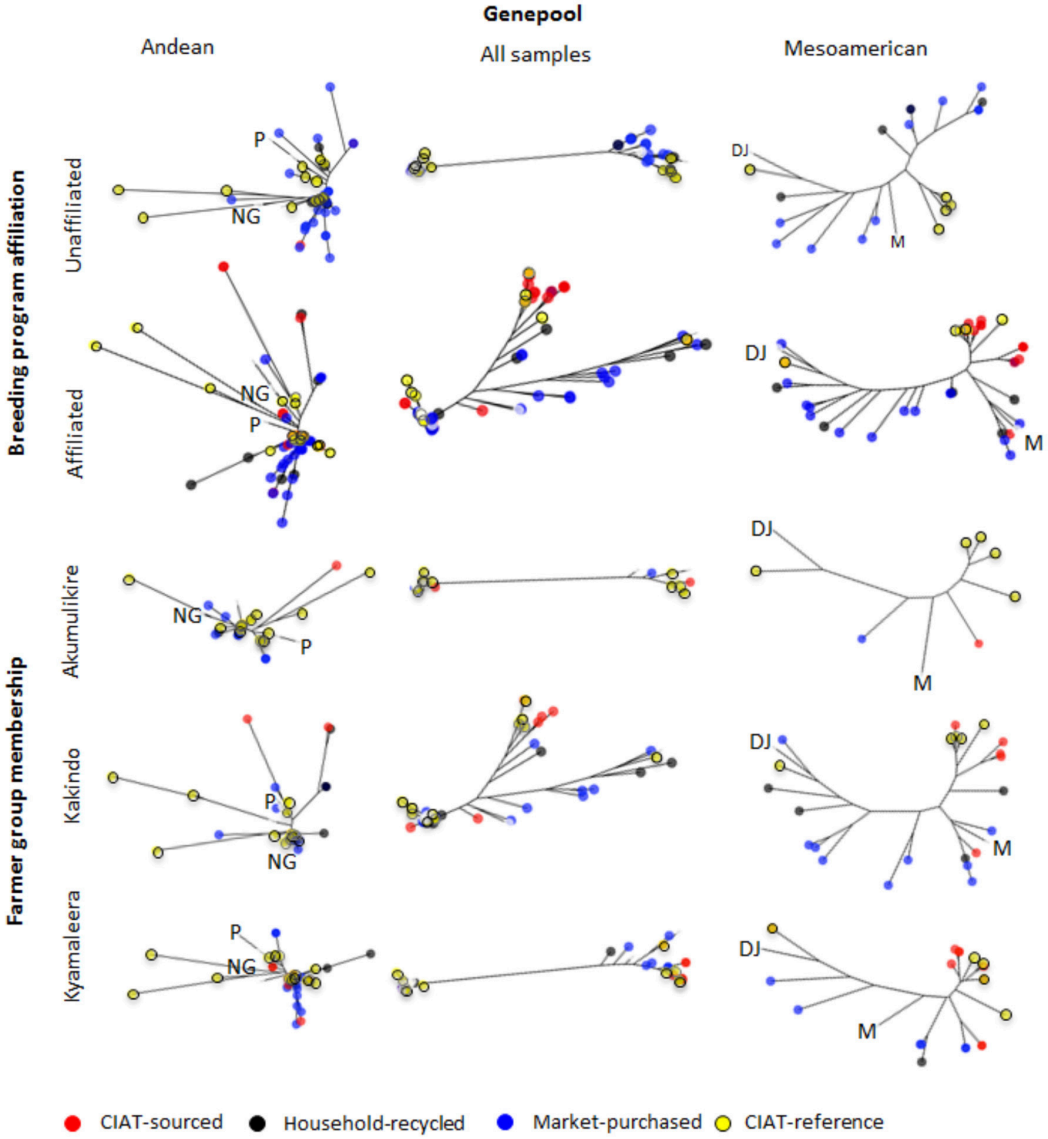
Neighbor-joining trees of single nucleotide polymorphism (SNP) diversity of Andean and Mesoamerican household and breeder-selected varieties between breeding program-affiliated and -unaffiliated households and across farmer groups based on Nei's genetic distance. Control genotypes of Peru (“P”), and Nueva Granada (“NG”) races of the Andean genepool and races Mesoamerican (“M”) and the Durango-Jalisco (“DJ”) complex of the Mesoamerican genepool are included as references. Bootstrap percentages (BP) were calculated for each node after 1,000 resamplings and all nodes with bootstrap values <70%, were collapsed. Branch node colors indicate seed source; CIAT-sourced (red), household-recycled (black), market-purchased (blue). CIAT-reference varieties are also depicted (yellow).

Neighbor-joining trees indicate that breeding program-affiliated households adopted Mesoamerican varieties (red dots in Figure 6) that were distinct from market-purchased (blue dots) and household-saved (black dots) seed stocks. One third (five samples) of the CIAT-sourced Mesoamerican household seed samples were assigned a potential breeder-selected source variety with a reasonable degree of confidence. NABE2 was a potential breeder-selected source Mesoamerican variety among Kyamaleera and Kakindo households (Tables 1, 4). One sample of K 131 and one of Roba 1 were also present among the CIAT-sourced samples from Kyamaleera households.

## Discussion

### Household crop representativeness and seed Engufu as novelty

In spite of the relatively small area covered by the Hoima region, household seed stocks contained a fairly representative collection of both Andean and Mesoamerican material (Figures 1, 2). The household collection also contained seed that was distinct from material found in the most recent and extensive collection of common bean germplasm available at the time of the study.

This assessment determined that Seed Engufu, while embedded within the Andean group, was not only molecularly monomorphic and distinct from other household samples but also from all the samples included in the Andean Diversity Panel and the Core Collection from the USDA (Figures 1, 2). Seed Engufu was also represented as a pure line in the STRUCTURE analysis, which further distinguished household samples from the improved varieties. The latter displayed mixed genetic background from crossing events in breeding programs. Four samples from the Andean Diversity Panel showed high levels of genetic relatedness to Seed Engufu, providing candidates for likely relatives of Seed Engufu. These samples that were all collected from eastern Africa (Rozi Koko or Rose Coco, PI449428, ADP751 and ADP758). PI449428 (local name: Koko) was provided by H. Van Rheenen to the USDA Regional Plant Introduction Station (Pullman, WA) in 1980 (GRIN-Global, http://npgsweb.ars-grin.gov). PVS-trial varieties that were evaluated by breeding program-affiliated households did not show high levels of genetic relatedness to Seed Engufu. K20 and K 132 were the most morphologically similar to Seed Engufu of all the varieties included in the PVS-trials. Both varieties are Calima-type improved material that were released and disseminated with widespread adoption in Uganda over the last three decades (Table 1). K20 was released in 1968 as the first product of bean research activities and was still widely grown in Uganda when Grisley and Mwesigwa (1994) evaluated bean production in 1994. That same year, the Uganda National Bean Program released K 132, a Calima seed type similar to K20 (David et al., 2000). By 1998, K 132 was sown on an estimated 4,100 ha with a production increase of 290 tons (David et al., 2000). The historical popularity of these widely adopted varieties suggests that these were likely candidates for Seed Engufu. However, Seed Engufu was shorter than K20 and K 132 and, at the molecular level, belonged to a different population than Seed Engufu samples. The individual membership coefficient estimates of K20 and K 132 do, however, indicate that Seed Engufu forms the genetic background of these breeder-selected samples. Forty percent of the genetic composition of K20 samples and 24% of K 132 was of the Seed Engufu population cluster (*K* = 4). K20 might have the highest proportion of *K* = 4 membership because it is a product of the earliest breeding effort of the breeder-selected samples in this study.

### Seed systems and local varietal diversity

This study takes a unique approach, linking household seed source data to seed stock molecular analysis, to explain how household seed sourcing activities impact crop genetic diversity and maintenance of novel germplasm. Studies like this are increasingly relevant as the importance of crop genetic diversity for household producers gains wider recognition. For instance, Jarvis and Hodgkin (2008) argued that varietal mixtures represent a major form of insurance against losses when production constraints are variety specific. Diversity has also been associated with productivity of agro-ecosystems and health of ecosystem services (Ceroni et al., 2007; Hajjar et al., 2008), such as disease and pest control (Abate et al., 2000), which can decrease the need for agricultural inputs like fertilizers and pesticides and, as a result, limit the negative consequences associated with their use (Tilman et al., 2002). Thus, diversification during initial stages of adoption can represent desirable outcomes of agricultural development practice.

At the same time, there is growing concern over the loss of traditional varieties, like Seed Engufu, the unique Calima type found among the producers in this study. Concern that traditional varieties would be rapidly replaced by improved varieties emerged in the 1970s and 1980s as evidence grew to support this claim (Frankel and Soulé, 1981). Recent studies have, however, suggested that rural household producers have maintained or increased use of traditional materials in their production systems (Chambers et al., 2007; Bezançon et al., 2009). Other studies have found that household adoption of improved varieties can support conservation of local genetic diversity, representing two mutually supportive processes. For instance, an evaluation of common bean planted as varietal mixtures among household producers in the Kivu region of Zaire, Trutmann and Pyndji (1994) found that local bean varietal mixtures were more productive when 25% of the mixture was of improved, angular-leaf-spot-resistant varieties, BAT76 or A285. The increased yields associated with partial adoption had potential to provide enough seed for sales and consumption, allowing households to save and maintain local crop diversity rather than sell or eat local and diverse seed stocks.

The comparisons between adopted breeder-selected materials and market-sourced or household-saved seeds from the current study revealed a range of seed acquisition patterns that varied in levels of adoption, diversification, and replacement of Seed Engufu. These patterns show that adoption can lead to a wide range of outcomes. In certain cases, adoption diversified household seed stocks but resulted in partial replacement of the novel landrace, Seed Engufu. In other cases, market-sourced and household-saved material supported higher levels of diversity and maintenance of novel landraces than adopted material. These results show that outcomes, and potential benefits, of adoption can vary across groups of households within a relatively small geographic area.

The long-term consequences of adoption and diversification (e.g., on-farm yields, contribution to household food availability, and income) from this study remain unresolved. In 2013, at the end of the participatory varietal selection (PVS) trials, households ranked the local varieties “Masindi Yellow Long” and Seed Engufu among the top three most preferred varieties of PVS trials varieties—favoring them over all of the breeder-selected varieties. Masindi Yellow and Seed Engufu were still the most preferred in 2016 (C. Mukankusi, unpubl. results). According to informal household interviews that were conducted during seed collections, the traits that households associated with breeder-selected samples included high yields, pest-resistance, and good taste. Alternatively, the traits that households associated with household-saved varieties included early maturity, drought resistance and link to family heritage. These accounts highlight a disconnect between the traits under selection by formal-seed-sector breeders and household producers. They also suggest that both formal breeders and household producers can complement one another by providing germplasm with distinct types of desirable traits.

The morphological and molecular traits that characterized Seed Engufu suggest that it has potential as a valuable source of genetic material for common bean improvement for household production in the Hoima region of Uganda and other regions with similar agronomic and social conditions. Landraces are generally regarded as valuable sources of genetic variability based on the assumption that they are selected under a wide range of biotic and abiotic stresses found on farmers' fields while the high yield capacity for which improved cultivars were bred was limited to a set of ideal growing conditions (Zeven, 1998). However, phenotypic evaluation of Seed Engufu performance is still needed to evaluate these samples and inform decisions on how to incorporate Seed Engufu in future production, dissemination and breeding efforts. A comparison of household-saved, market-purchased and organization-sourced seed performance and longitudinal production and market surveys is also needed to better understand consequences of seed acquisition and adoption.

### Role of seed exchange networks

**Figure 7:**
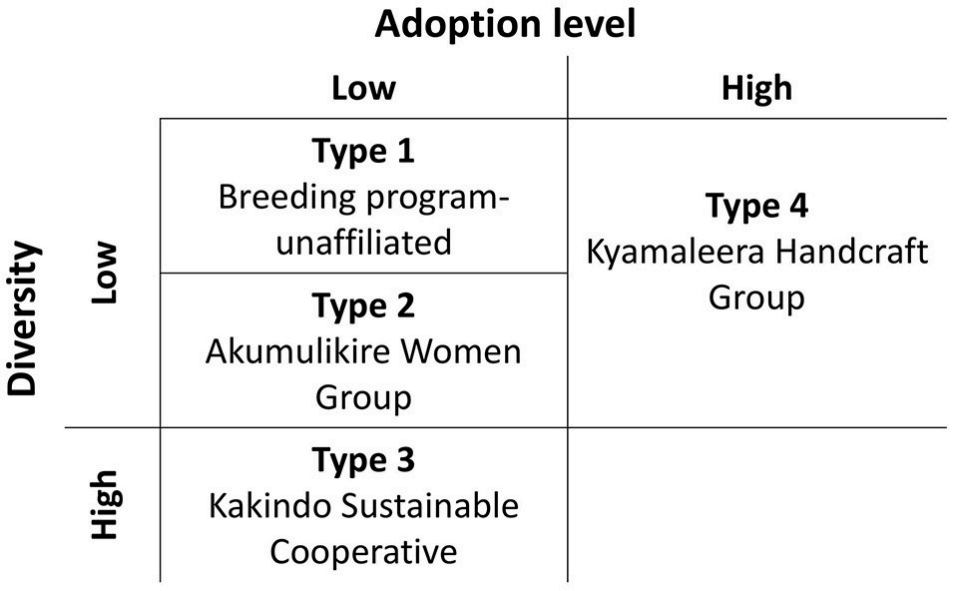
Adoption types of household producers in Hoima, Uganda. Types vary in levels of adoption (high, low), and seed stock diversity (high, low). Maintenance and investment in Seed Engufu are represented as four Types along these dimensions. High (Type 1) and low (Type 2) levels of both household-saved and market purchased Seed Engufu. A high level of household-saved Seed Engufu and a low level of market purchased Seed Engufu (Type 3). Partial replacement of household-saved Seed Engufu (Type 4).

Distinct adoption patterns, described on the basis of the overall household seed stock characteristics and the consequences that adopted material had on that seed stock, emerged among the four groups of households (Figure 7). Consequences of adoption for household seed stock composition and diversity depended on the contributions of alternative seed sources (market-sourced and household-saved) to household seed stock. The four distinct adoption patterns varied in levels of adoption (Table 2), seed stock diversity (Table 4), total molecular variance (Table 5), and maintenance and investment in Seed Engufu, a local Calima-type landrace of the Andean genepool (Figure 7) as presented in the next paragraphs.

**Table 4:** Genetic diversity of household seed stock expressed as standardized molecular variance within household groups.

	**Between affiliated and unaffiliated households**			**Across affiliated farmer groups**			
	**Unaffiliated Mean**	**Affiliated Mean**	**Fixation index *Φ_*PT*_*^a^**	**Akumulikire**	**Kakindo**	**Kyamaleera**	**Fixation index *Φ_*PT*_***
**ALL**							
All	606.14^b^	774.93	0.015	481.45	877.90	764.45	0.021
A	**165.49**	**183.45**	0.032^*^	**162.21**	**188.02**	**177.03**	0.047^*^
M	**495.47**	**566.30**	0.067^*^	544.00	611.03	506.96	0.029
**ORGANIZATION**							
All	244.50	826.87	−0.027	712.25	1003.38	747.41	0.059
A	244.50	155.27	0.187	359.5	277.08	123.07	0.058
M	0.00	434.18	–	–	447.55	439.28	0.017
**HOUSEHOLD**							
All	603.63	858.82	0.001	–	941.54	802.67	−0.057
A	140.72	233.62	0.016	–	163.73	268.83	0.300
M	533.5	634.95	−0.062	–	641.75	–	–
**MARKET**							
All	621.02	699.77	−0.010	360.79	784.20	786.22	0.014
A	159.43	160.69	−0.002	136.64	168.24	162.50	0.031
M	504.11	594.63	0.007	–	604.40	565.67	0.064
**FIXATION INDEX ACROSS SOURCES WITHIN FARMER GROUPS, *Φ_*PT*_***							
All	−0.039	0.018		0.178	0.249	−0.003	
A	−0.012	0.143^*^		0.265	0.105	0.276^*^	
M	−0.079	0.786^*^		–	0.011^*^	0.050	

*Andean (A) and Mesoamerican (M) seed were aggregated and evaluated as distinct sub-populations for seed from All sources combined and each source separately. Analysis of molecular variance (AMOVA) partitioned standardized molecular variance within and across seed sources, within and between breeding program-affiliated and -unaffiliated households and within and across farmer groups to evaluate the percent of seed stock variability explained by seed source, breeding program-affiliation and farmer group-membership. Significance was calculated using 16,000 permutations*.

a *ΦPT is calculated as AP / (WP + AP) = AP / TOT where AP, Estimated variance among populations; WP, Estimated variance within populations*.

b *Genetic Diversity is Calculated as SSWP x (n−1)^−1^. Where SSWP, Within-population sum of squares and n, number of samples*.

“*” *means significance level of p < 0.05*.

*Numbers in bold: values that are significantly different between affiliated and unaffiliated households or across affiliated farmer groups*.

**Table 5:** Total molecular variance (MV) of household seed stock expressed as standardized molecular variance within household groups.

	**Between affiliated and unaffiliated households**			**Across affiliated farmer groups**			
	**Unaffiliated Mean**	**Affiliated Mean**	**Fixation index *Φ_*PT*_*^a^**	**Akumulikire**	**Kakindo**	**Kyamaleera**	**Fixation index *Φ_*PT*_***
**ALL**							
All	46,673	90,667	0.015	7,222	35,994	44,338	0.021
**A**	**10,095**	**15,043**	0.032^*^	**2,109**	**4,701**	**7,258**	0.047^*^
**M**	**7,432**	**19,254**	0.067^*^	544	9166	8,111	0.029
**ORGANIZATION**							
All	245	90,667	−0.027	1,425	8,027	23,170	0.059
A	245	15,043	0.187	360	831	2,707	0.058
M	0.00	19,254	–	–	1790	3,514	0.017
**HOUSEHOLD**							
All	11,469	12,024	0.001	–	8,474	2,408	−0.057
A	2,111	2,103	0.016	–	819	538	0.300
M	1,601	2,540	−0.062	–	1925	–	–
**MARKET**							
All	34,156	40,587	−0.010	3,969	17,252	17,297	0.014
A	6,855	6,910	−0.002	1,367	2,524	2,438	0.031
M	5,545	8,325	0.007	–	3,626	3394	0.064
**FIXATION INDEX ACROSS SOURCES WITHIN FARMER GROUPS, *Φ_*PT*_***							
All	−0.039	0.018		0.178	0.249	−0.003	
A	−0.012	0.143^*^		0.265	0.105	0.276^*^	
M	−0.079	0.786^*^		–	0.011^*^	0.050	

*Andean (A) and Mesoamerican (M) seed were aggregated and evaluated as distinct sub-populations for seed from All sources combined and each source separately. Analysis of molecular variance (AMOVA) partitioned total molecular variance within and across seed sources, within and between breeding program-affiliated and -unaffiliated households and within and across farmer groups to evaluate the percent of seed stock variability explained by seed source, breeding program-affiliation and farmer group-membership. Significance was calculated using 16,000 permutations*.

a *ΦPT is calculated as AP/(WP + AP) = AP / TOT where AP, Estimated variance among populations; WP, Estimated variance within populations Molecular variance (MV), Within-population sum of squares or SSWP*.

“*” *means significance level of p < 0.05*.

*Numbers in bold: values that are significantly different between affiliated and unaffiliated households or across affiliated farmer groups*.

*Type 1. Breeding program-unaffiliated: Low levels of adoption with low levels of seed stock diversity and high levels of household-saved (maintenance) and market-purchased (investment in) Seed Engufu*.

Breeding program-unaffiliated households displayed low levels of adoption (Table 2) and relatively low levels of overall genetic diversity (Table 4) in their seed stocks. There was no evidence of adopted Mesoamerican samples among unaffiliated household seed stocks. Although adoption and seed stock genetic diversity was low, evidence of Seed Engufu, a molecularly distinct and local Calima-type landrace of the Andean genepool, was common among PVS–unaffiliated households. Multiple seed sources contributed to the relatively high prevalence of Seed Engufu in household seed stocks. Breeding program-unaffiliated households had maintained Seed Engufu by saving household seeds from previous seasons and had invested their financial capital through market purchases of Seed Engufu.

*Type 2. Akumulikire Women Group: Low levels of adoption with low levels of seed stock diversity and the rare occurrence of Seed Engufu*.

Akumulikire Women Group households had the lowest levels of Mesoamerican seed adoption of the three breeding-program-affiliated farmer groups (Table 2). One sample (5%) from the entire collection of household seed stock represented adopted Mesoamerican material. Andean seed adoption was also uncommon among the Akumulikire households where adopted Andean seed represented just 9% of household seed stocks. Overall genetic diversity was lowest among members of Akumulikire Women Group [Sum of squares within-populations (SSWP): 481.45, Table 4], below that of the PVS-unaffiliated households (SSWP: 606.14, Table 4). Household-saved seed contributed particularly low levels of genetic diversity since only one household-saved Andean sample was present in the entire seed collection from the Group. The single Mesoamerican sample found among the Akumulikire Women Group households was CIAT-sourced and closely related to NABE 3 (Figure 6). This evidence suggests that adoption of a breeder-selected variety (most likely NABE 3), was necessary for Mesoamerican material to be represented among households from the Akumulikire Women Group. Finally, only three samples of Seed Engufu were identified in seed stock from Akumulikire Women Group member households (Figure 7). Surveys indicate that all three samples were sourced from the market, suggesting that the presence of Seed Engufu in this Group depended entirely on household market access. Genetic diversity of adopted Andean seed stock was highest among Akumulikire households (SSWP: 359.5, Table 4) even though adopted Andean seed was uncommon in this group (9%: Table 2). Due to this high level of diversity, the three samples of adopted Andean samples, diversified the otherwise, very uniform Andean seed stock of Akumulikire households.

*Type 3. Kakindo Sustainable Cooperative: Low levels of adoption with high levels of seed stock diversity and high levels of household-saved Seed Engufu*.

Households from the Kakindo Sustainable Cooperative displayed low levels of adoption (12%, Table 2). However, overall genetic diversity of the Kakindo Sustainable Cooperative household seed stock was the highest of all the groups. The Kakindo seed stock contained the largest proportion of household-saved Andean seed (15%: Table 2) and provided the only evidence of household-saved Seed Engufu (Figure 7). In contrast, households outside of Kakindo Sustainable Cooperative depended entirely on the market for Seed Engufu. Furthermore, household purchases and seed saving of rare Mesoamerican bean varieties diversified and maintained novel genetic material (Figure 7). Market-sourced Mesoamerican beans were particularly abundant among these households, contributing relatively high levels of genetic diversity (SSWP: 604.40, Table 4). Household-saved Mesoamerican seeds also contributed high levels of genetic diversity to the overall seed stock (SSWP: 641.75, Table 4). Kakindo Sustainable Cooperative households also adopted rare Mesoamerican seed samples representative of a unique Mesoamerican clade (Figure 6). Although adopted Mesoamerican samples were more common in seed stocks of Kyamaleera Handcraft Group members, Kakindo Sustainable Cooperative members adopted the most diverse set of Mesoamerican seeds of all the farmer groups (SSWP: 611.03, Table 4). The adopted Mesoamerican seed was closely related to breeder-selected varieties: K 131, NABE 2, and NABE 3, demonstrating that seed stock among breeding-program-affiliated household contained novel genetic material within race Mesoamerica as a result of adopting Mesoamerican varieties.

*Type 4: Kyamaleera Handcraft Group: High levels of adoption with high levels of seed stock diversity and partial replacement of Seed Engufu*.

Households from Kyamaleera Handcraft Group, displayed high levels of adoption where organization-sourced Andean samples were more common in seed stock from this group (35 ± 33) compared to Kakindo- (9 ± 20) and Akumulikire- (6 ± 0.13) households, *F*_(2, 40)_ = 6.11, *p* = 0.0050 (Table 2). Adopted and market purchased Andean seed were equally represented in seed stock of Kyamaleera Handcraft Group member households. In contrast, market-sourced seeds represented over 70% of seed stock of breeding program-unaffiliated households, members of the Akumulikire Women Group and members of Kakindo Sustainable Cooperative.

Kyamaleera households tended to adopt Andean material with higher levels of admixture between Seed Engufu and Nueva Granada races compared to the Andean material adopted by Akumulikire Women Group and Kakindo Sustainable Cooperative households (Figure 7). Despite the genetic background material represented in adopted samples from Kyamaleera households, neighbor-joining trees of Andean samples indicate that the adopted Andean seed samples from Kyamaleera Handcraft Group households were all closely related to the household-saved and market-sourced seed stocks (Figure 6). These adoption patterns suggest that Kyamaleera Handcraft Group members limited adoption to more familiar materials. Still, one household from the Kyamaleera Handcraft Group provided the only evidence of Roba 1 adoption.

This study provides evidence that seed exchange networks can have major implications for seed acquisition, crop population genetics, and maintenance of distinct varieties. Households that participated in PVS trials showed higher levels of molecular variance overall, which was due principally to acquisition of seeds from organizations, highlighting the importance of PVS trials (Table 4). The presence of NABE15 and NABE17 as potential breeder-selected sourced Andean varieties among the breeding program-affiliated households indicated preferential adoption of these varieties. These same households were clearly more likely to adopt rare Mesoamerica varieties and maintain more diverse seed stock than households that had not participated in the breeding activities. Identification of NABE 2, K 131 and Roba 1 in household seed stock cannot be interpreted as a preference for these Mesoamerican varieties because only one third (five samples) of the CIAT-sourced Mesoamerican household seed samples were identified with a reasonable degree of confidence (genetic distance of less than 0.1). The higher level of relatedness between CIAT-sourced household samples and breeder-selected varieties of Andean samples compared to Mesoamerican samples might reflect a more stringent selection for preferred varieties (i.e., Andean) than for varieties seen as Supplementary (i.e., Mesoamerican). In addition to the heterogeneity of the released Mesoamerican seed material, Mesoamerican varietal adoption by the breeding program-affiliated households in this study might have been subject to more relaxed selection and a greater likelihood of mechanical mixture or spontaneous outcrossing than Andean adoption.

Evidence of adopted Mesoamerican varieties in household seed stocks further challenges the conventional assumption that formal seed systems have a limited or inconsequential role, relative to local seed systems, in shaping the crop varieties grown by household producers. This assumption has been largely based on evidence that households from eastern and central Africa sourced the majority of their seed from local seed channels (farmer-saved, farmer-to-farmer exchange, and local markets) rather than the formal seed sector (David and Sperling, 1999). However, more recent, econometric models of adoption have found that the relative importance of community- and regional-level seed networks can vary across countries (Thuo et al., 2014). For instance, adoption by households from Kenya depended mainly on household access to external support services (research and extension services). The current study found that both regional and local seed systems explained household adoption patterns in western Uganda where breeding programs—PVS trials in particular—were well established.

Differences between breeding program-affiliated and -unaffiliated households suggest that household participation in PVS trials can represent a main factor in household seed acquisition and seed stock management processes. The variability that was observed across farmer groups suggests that this relatively localized type of seed exchange network can also represent a main factor underlying these processes. These findings suggest that involvement in regional-level breeding programs and local farmer groups can drive seed acquisition and management decisions by household producers. However, additional studies are needed to identify the components and dynamics of regional and local seed systems that shape decision-making among household producers. These studies can help support efforts that promote adoption without compromising maintenance of novel genetic material, including potential landraces like Seed Engufu.

## Ethics statement

The study (IRB application ID 522529-1) conformed to the principles of sound research ethics and was designated exempt (2) research, in accordance with the UC Davis Institutional Review Board (IRB). All participants and local tribal authorities were provided with key elements of informed consent and gave verbal consent to participation in the study. A Memorandum of Understanding (MoU) also established support from research partners and local authorities including the Kyabigambire Rural Integrated Development Association (KRIDA) and the Hoima District Government.

## Author contributions

CM was involved in the participatory varietal selection that initiated this study, EW and PG designed the research presented here, including the family surveys and genetic diversity studies, EW conducted the research in Uganda and at UC Davis, JB assisted in the genetic analyses, EW wrote the first draft, and all authors edited the subsequent versions of the manuscript.

### Conflict of interest statement

The authors declare that the research was conducted in the absence of any commercial or financial relationships that could be construed as a potential conflict of interest.
